# *miR-494* up-regulates the PI3K/Akt pathway via targetting PTEN and attenuates hepatic ischemia/reperfusion injury in a rat model

**DOI:** 10.1042/BSR20170798

**Published:** 2017-09-19

**Authors:** Song Su, De Luo, Xiangdong Liu, Jiang Liu, Fangyi Peng, Cheng Fang, Bo Li

**Affiliations:** Department of Hepatobiliary Surgery, The Affiliated Hospital of Southwest Medical University, Luzhou City 646000, Sichuan Province, P.R. China

**Keywords:** apoptosis, AML12 cells, hepatic ischemia/reperfusion injury, MicroRNA-494, PI3K/Akt

## Abstract

A rat HIRI model was constructed and treated with an intraperitoneal injection of agomir-*miR-494* or agomir-NC (negative control) for 7 days after the surgery. The pathophysiological changes in sham-operated rats, HIRI, HIRI + agomir-*miR-494*, and HIRI + agomir-NC were compared. The effect of *miR-494* was also assessed in an H_2_O_2_-induced apoptosis model. Hepatic AML12 cells were transfected with mimics NC or *miR-494* mimics, followed by 6-h H_2_O_2_ treatment. Cell proliferation and apoptosis were detected by CCK8 assay and flow cytometry, respectively. Further, the *miR-494* target gene was identified by luciferase reporter assay, and verified both *in vitro* and *in vivo* experiments. The activity of AKT pathway was further analyzed *in vivo* by Western blot. HIRI + agomir-*miR-494* rats exhibited significantly higher *miR-494* expression, lower serum alanine aminotransferase (ALT), aspartate aminotransferase (AST), lactate dehydrogenase (LDH), and glutamate dehydrogenase (GLDH) level, lower hepatic MDA, TOA, and OSI, alleviated hepatic necrosis, reduced hepatocyte apoptosis, and decreased expression of apoptosis-related proteins, when compared with HIRI + agomir-NC rats (*P*<0.05 or 0.01). After H_2_O_2_ treatment, AML-12 cells transfected with *miR-494* mimics had significantly higher proliferation and lower apoptosis rate compared with mimics NC group (*P*<0.01). PTEN was identified as an *miR-494* target gene. PTEN expression was significantly down-regulated in AML12 cells transfected with *miR-494* mimics, and was up-regulated by treatment of *miR-494* inhibitor (*P*<0.01). Moreover, HIRI + agomir-*miR-494* rats exhibited significantly lower PTEN expression, and higher p-AKT, p-mTOR, and p-p70S6K levels compared with HIRI + agomir-NC rats. Therefore, *miR-494* protected rats against hepatic ischemia/reperfusion (I/R) injury through down-regulating its downstream target gene *PTEN*, leading to the activation of PI3K/AKT signaling pathway.

## Introduction

Hepatic ischemia/reperfusion (I/R) injury is an inevitable complication occurring during surgical procedures such as partial hepatectomy and liver transplantation [1,2]. During hepatic I/R injury, high levels of reactive oxygen species (ROS) are produced, leading to acute inflammatory responses and hepatocyte apoptosis, which ultimately result in liver dysfunction and even liver failure [3–8].

miRNAs are a class of small non-coding RNAs (21–25 nts) that suppress gene expression by binding to the 3′-UTR of the target mRNA [9], and therefore are associated with a variety of biological processes, such as cell differentiation, proliferation and apoptosis, as well as the occurrence and development of diseases [10]. Several studies have suggested the important roles of miRNAs in I/R injury, such as *miR-122, miR-124, miR-146a, miR-223, miR-370* [11–15]. Differentially expressed *miR-494* has been reported in rats with cerebral I/R injury [16]. *miR-494* alleviates the I/R-induced myocardium injury in a mouse model through activating the AKT signaling pathway [17]. Overexpression of *miR-494* exerts protective effects against hypoxia/ischemia-induced apoptosis in human hepatic L02 cells via the modulation of PI3K/Akt pathway [18]. However, the potential *in vivo* role of *miR-494* in hepatic I/R injury remains unknown. Therefore, the current study was undertaken to investigate the effect and relevant molecular mechanism of *miR-494* in response to I/R-induced hepatic injury in a rat model. We further validated our findings in hepatic AML12 cells treated with H_2_O_2_-induced stress. Our study may provide new insights into the development of novel therapeutic strategies for hepatic I/R injury.

## Materials and methods

### Construction of a rat HI/RI model

Male Sprague–Dawley (SD) rats weighing 180–220 g were purchased from the Slac Laboratory Animals (Shanghai, China) and housed in standard conditions with free access to water and food before this experiment. SD rats were randomly divided into sham, HIRI, HIRI + agomir-NC (negative control), and HIRI + agomir-*miR-494* groups (*n*=6 in each group). Rats in HIRI group were anesthetized by intraperitoneal injection of 3 ml/kg chloral hydrate. Partial (70%) hepatic ischemia was introduced by clamping the artery and portal venous blood supply to the middle and left liver lobes with atraumatic vascular clamps. After 60-min ischemia, the clamp was removed and the liver was reperfused for 6 h. The sham group underwent abdominal surgery without liver I/R. Rats in HIRI + agomir-*miR-494* were given an intraperitoneal injection of 20 μl of 500 pmol agomir-*miR-494* (GenePharma Biotech, Shanghai, China)/day for 7 days prior to ischemia. Rats in HIRI + agomir-NC were given equal amount of agomir-NC. Rats were immediately killed after the surgery, and blood and left liver samples were collected. The present study was approved by the Research Ethics Committee and performed in strict accordance with the institutional animal care instructions.

### Liver enzyme assay

Indexes of liver injury including serum activities of alanine aminotransferase (ALT), aspartate aminotransferase (AST), lactate dehydrogenase (LDH), and glutamate dehydrogenase (GLDH) were determined using commercial kits (Jiancheng Bioengineering Institute, Nanjing, China) following the manufacturer’s instructions.

### Quantitative reverse-transcription PCR

Liver tissue was homogenized on ice. Total RNA was extracted using the TRIzol reagent (Invitrogen, Shanghai, China), and reverse-transcribed into cDNA using SuperScript II (Invitrogen). Real-time PCR was then performed using SYBR Green PCR Master Mix (TaKaRa, Qingdao, China) in an ABI 7500 System (Applied Biosystems, Foster City, CA, U.S.A.). The following primers were synthesized by Invitrogen and used in the PCR: *miR-494*-F, 5′-TGGTGATGGGATTTGAAACATACACGGGAAAC-3′, *miR-494*-R, 5′-AGATAGACGG-TGTCGCTGTTGAAGTCAG-3′; U6-F, 5′-GCTTCGGCAGCACATATACTAAAAT-3′, U6-R, 5′-CGCTTCACGAATTTGCGTGTCAT-3′; PTEN-F, 5′-TGGAAAGGGACGAACTGGTG-3′, PTEN-R, 5′-CATAGCGCCTCTGACTGGGA-3′. The experiment was repeated three times. Data were analyzed using the 2^−Δ*C*^_t_ method. The relative expression of *miR-494* was calculated using small nuclear RNA U6 (snU6) as the internal control.

### Histological examination

Liver samples were fixed in 4% buffered formalin, dehydrated in graded ethanol (70, 80, 90, and 100%), embedded in paraffin, and cut into 4-μm sections. Tissue sections were then dewaxed with xylene, rehydrated in graded ethanol (100, 90, 80, and 70%), and stained with Hematoxylin and Eosin (HE). The pathological changes including inflammatory infiltration and hepatic cell necrosis in five randomly selected visual fields was observed.

### TUNEL apoptosis assay

Paraffin sections were dewaxed and hepatic cell apoptosis was detected by terminal deoxynucleotidyl transferase mediated dUTP nick-end labeling (TUNEL) assay using the TUNEL assay kit (Roche, Shanghai, China) according to the manufacturer’s protocol. Nuclei were labeled by DAPI staining (Biohao Biotech, Shanghai, China). Sections were then observed under a confocal laser microscope (Olympus, Japan). The percentage of apoptotic cells was calculated by counting TUNEL-positive cells in five randomly selected fields on each slide.

### Western blot

Total protein was extracted from harvested liver tissues or cells using lysis buffer (Beyotime, Shanghai, China) and quantitated using BCA Protein Assay Kit (Beyotime) following the manufacturer’s instructions. Protein samples were then separated by 10% SDS/PAGE and transferred on to PVDF membranes (GE Healthcare, Amersham, U.K.). Membranes were blocked with 5% skim milk and incubated with appropriate dilutions of primary antibodies overnight at 4°C as follows: rabbit anti-rat Bax, cleaved caspase-3, cleaved PARP, PTEN, AKT, p-AKT, mTOR, p-mTOR, p70S6K, p-p70S6K, and β-actin. Membranes were then washed with PBST and incubated with goat anti-rabbit HRP–conjugated IgG at room temperature for 1 h. Immunoreactivity was detected using the ECL detection system (GE Healthcare). All antibodies were purchased from Abcam (Cambridge, MA, U.S.A.) or Cell Signaling Technology (Danvers, MA, U.S.A.). β-actin was used as the internal control.

### DNA fragmentation assay

The cytoplasmic histone-associated DNA fragmentation in different groups was determined using an ELISA Kit (Roche, Indianapolis, IN, U.S.A.). The experiment was performed in triplicate.

### Prediction of *miR-494* targets

Both PicTar (http://pictar.mdc-berlin.de) and TargetScan version 6.2 (http://www.target-scanorg/index.html) were used to identify potential downstream targets of *miR-494*. Genes that were predicted by both databases were considered as potential targets. PTEN, one of the potential targets was selected for further analysis.

### Luciferase reporter assay

The *PTEN* gene was analyzed using the online tool (http://www.targetscan.org) to predict the 3′-UTR sequence for *miR-494*. The oligonucleotide sequences (3′-UTR of PTEN wild and mutated type) were cloned into the site of a firefly luciferase reporter vector pMIR (Promega, WI, U.S.A.). HEK293T cells at 70% confluence were cotransfected with 500 ng of pMIR-PTEN-wt/pMIR-PTEN-mut and 50 nM of *miR-494* mimics/mimics C,G(enePharma Biotech) using a Lipofectamine 3000 transfection kit (Invitrogen). After 24 h, the luciferase activity was measured using a dual luciferase reporter assay system (Promega) following the manufacturer’s instructions. The relative luciferase activity (RLA) was calculated as the ratio of firefly luciferase activity (Ff) to *Renilla* luciferase activity (Rn).

### Cell culture and transfection

Murine hepatic cell line AML12 cells (ATCC, Manassas, VA, U.S.A.) were cultured in DMEM/Ham’s F12 medium supplemented with 10% FBS, 100 u/ml penicillin, 100 μg/ml streptomycin, 5 μg/ml insulin, 5 μg/ml transferrin, 5 ng/ml selenium, and 40 ng/ml dexamethasone. AML12 cells at 70% confluence in six-well plates were transfected with 50 nM of *miR-494* mimics, *miR-494* inhibitor, mimics NC, or inhibitor NC (GenePharma Biotech) using Lipofectamine 3000 transfection agent. After 48 h, total RNA and protein were extracted, and *PTEN* mRNA and protein expression was analyzed by quantitative reverse-transcription PCR (qRT-PCR) and Western blot as described above.

### H_2_O_2_-induced oxidative stress

AML12 cells at 70% confluence were treated with H_2_O_2_ (0, 25, 50, 100, 200, 300, and 400 μM) for 6 h. Cells were collected and *miR-494* level was measured by qRT-PCR as described above.

### CCK8 detection of cell viability

AML12 cells at 70% confluence were transfected with 50 nM of *miR-494* mimics or mimics NC. At 24, 36, and 48 h after transfection, cells were treated with 200 μM H_2_O_2_ for 6 h. CCK8 (Beyotime, 10 μl) was added to the cells, and the viability of the cells was measured at 450 nm using a microplate reader.

### Annexin V-FITC apoptosis detection

Transfected AML12 cells were treated with 200 μM H_2_O_2_ for 6 h. Cells were then collected and resuspended in 500 μl of binding buffer. Annexin V-FITC (5 μl) was added and cell apoptosis was detected by flow cytometry within 30 min.

### Detection of apoptosis-related proteins

Transfected AML12 cells were treated with 200 μM H_2_O_2_ for 6 h. The expression of cle-PARP, cle-caspase-3, AKT, and p-AKT was determined by Western blot as described above.

### Immunohistochemical analysis of apoptosis in liver tissue

Paraffin-embedded sections were dewaxed with xylene, and dehydrated with ethanol. Slices were processed to remove endogenous peroxidase, followed by antigen retrieval and serum blocking. The sections were incubated successively with rabbit anti-rat cleaved caspase-3, biotin-labeled secondary IgG, and horseradish peroxidase labeled streptavidin. Yellow or brown stained cells were positive for cleaved caspase-3.

### Detection of AKT signaling activity

Liver tissues collected from sham, HIRI, HIRI + agomir-NC, and HIRI + agomir-*miR-494* groups (*n*=6) was homogenized. The expression of PTEN, AKT, p-AKT, mTOR, p-mTOR, p70S6K, and p-p70S6K were analyzed by Western blot analysis as described above.

### Statistical analysis

All experiments were performed in triplicate. Data were expressed as mean ± S.D. All statistical analyses were performed using SPSS 16.0 software (IBM SPSS, Chicago, IL, U.S.A.). Differences between two groups were determined by the Student’s *t* test. Differences amongst groups were compared using one-way ANOVA followed by post-hoc Tukey HSD test. *P*<0.05 was considered as statistically significant.

## Results

### I/R led to injury of rat liver through induction of oxidative stress and apoptosis

**Figure 1:**
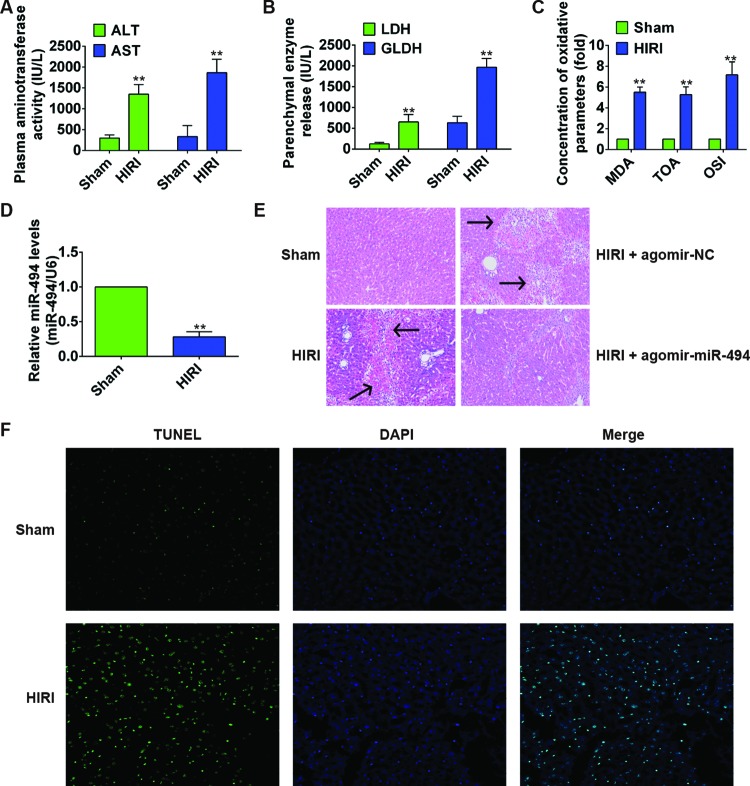
*miR-494* protected against liver I/R injury in SD rats SD rats (*n*=6 rats per group) were sham-operated or subjected to 60-min liver ischemia followed by 6-h reperfusion. HIRI rats were treated with agomir-*miR-494* or agomir-NC. (**A**,**B**) Serum level of ALT and AST, LDH, and GLDH was determined using commercial kits. (**C**) Concentration of liver oxidative parameters MDA, TOA, and OSI. (**D**) Relative *miR-494* level in liver samples from different groups was measured by qRT-PCR. *miR-494* expression was normalized to U6. (**E**) HE staining of liver samples. Arrow indicates hepatocyte injury. (**F**) TUNEL staining of hepatocellular apoptosis. All experiments were performed in triplicate, and data were represented as mean ± S.D. *, *P*<0.05 and **, *P*<0.01 compared with sham surgery group.

To investigate the potential role of *miR-494* in liver I/R injury, we constructed a rat HIRI model. As shown in Figure 1A,B, after the liver I/R surgery, serum level of liver injury indexes including ALT, AST, LDH, and GLDH enzymes was significantly increased compared with sham surgery group (all *P*<0.01), revealing that I/R induced liver injuries. Moreover, the concentration of oxidative parameters MDA, TOA, and OSI was markedly increased in HIRI group (Figure 1C). HE staining revealed loss of cell integrity, large areas of hepatocyte necrosis, and inflammatory infiltration in the livers in HIRI group (Figure 1E). We further evaluated the extent of apoptosis in livers by TUNEL assay, and found that the number of TUNEL-positive cells in HIRI group was notably higher compared with sham surgery group (Figure 1F). These pathophysiological changes suggested the successful construction of HIRI model in the present study. We then examined the expression levels of *miR-494*. As shown in Figure 1D, *miR-494* in HIRI group was significantly down-regulated at least two fold when compared with sham surgery group (*P*=0.007).

### *miR-494* attenuated hepatic I/R injury in rats

**Figure 2:**
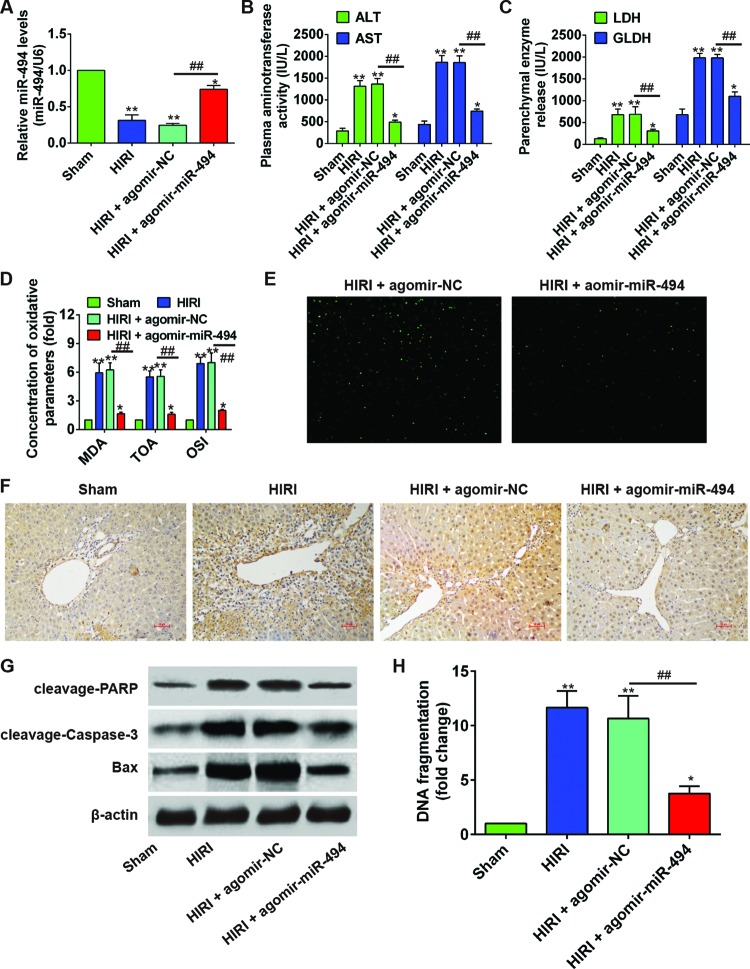
*miR-494* protected against liver I/R injury in SD rats SD rats (*n*=6 rats per group) were sham operated or subjected to 60-min liver ischemia followed by 6-h reperfusion. HIRI rats were treated with agomir-*miR-494* or agomir-NC. (**A**) Relative *miR-494* level in liver samples from difference groups was measured by qRT-PCR. *miR-494* expression was normalized to U6. (**B**,**C**) Serum level of ALT, AST, LDH, and GLDH was determined using commercial kits. (**D**) Concentration of liver oxidative parameters MDA, TOA, and OSI. (**E**) TUNEL staining of hepatocellular apoptosis. (**F**) IHC detection of cleaved caspase-3 in different groups. (**G**) Hepatic expression of apoptosis-related proteins cleavage PARP, cleavage Caspase-3, Bax were determined by Western blot using β-actin as an internal control. (**H**) DNA fragmentation assay was performed. All experiments were performed in triplicate, and data were represented as mean ± S.D. *, *P*<0.05 and **, *P*<0.01 compared with sham surgery group; ^ ##^, *P*<0.01 compared with agomir-NC group.

In order to analyze the regulatory role of *miR-494* in I/R injury, we treated the HIRI rats with an intraperitoneal injection of agomir-*miR-494* or agomir-NC. As shown in Figure 2A, although the liver *miR-494* concentration in HIRI + agomir-*miR-494* group was significantly lower than that in sham surgery group (*P*=0.046), it was substantially increased compared with HIRI + agomir-NC (*P*=0.004). Serum ALT, AST, LDH, and GLDH levels in HIRI + agomir-*miR-494* group were significantly decreased, as compared with HIRI + agomir-NC group (all *P*<0.01, Figure 2B,C). Likewise, oxidative parameters of the liver including MDA, TOA, and OSI was significantly reduced compared with agomir-NC treated group (all *P*<0.01, Figure 2D). Moreover, liver histology from HIRI + agomir-*miR-494* rats revealed obvious reduction in I/R-induced hepatocellular necrosis and improvement of cell integrity (Figure 1E). IHF demonstrated a markedly lower number of TUNEL-positive cells in HIRI + agomir-*miR-494* group (Figure 2E). Immunohistochemical (IHC) results demonstrated a substantially higher expression of cleaved caspase-3 in HIRI and HIRI + agomir-NC groups compared with sham group (Figure 2F). In contrast, the cleaved caspase-3 expression in HIRI + agomir-*miR-494* group was only slightly higher compared with sham group. Consistently, the hepatic levels of cleavage PARP, cleavage Caspase-3, and Bax in HIRI + agomir-*miR-494* group was clearly lower compared with agomir-NC group (Figure 2G). DNA fragment was significantly reduced in agomir-*miR-494* treated rats (*P*=0.001, Figure 2H). All together, these results suggested that *miR-494* effectively alleviated the I/R injuries in rat liver via inhibiting the cell apoptosis.

### *miR-494* decreased H_2_O_2_-induced apoptosis of hepatic AML12 cells by activating AKT

**Figure 3:**
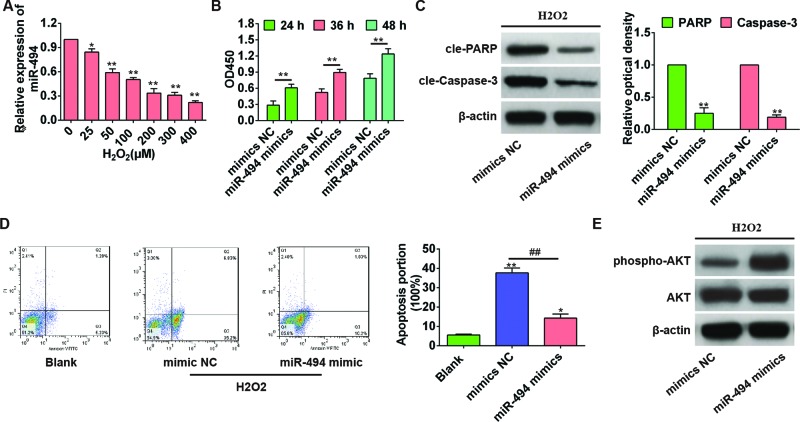
*miR-494* reduced the H_2_O_2_-induced apoptosis of hepatic AML12 cells by activating AKT *in vitro* (**A**) Hepatic AML12 cells were treated with H_2_O_2_ (0–400 µM) for 6 h, and the level of *miR-494* was evaluated by qRT-PCR using U6 as an internal control. *, *P*<0.05 and **, *P*<0.01 compared with control cells without H_2_O_2_ treatment. (**B**) AML12 hepatocytes were transfected with mimics NC or *miR-494* mimics for 24, 36, and 48 h, and then treated with 200 µM H_2_O_2_. Cell viability was measured by CCK8 assay. **, *P*<0.01 compared with mimics NC group. (**C**) Apoptosis-associated protein was detected by Western blot. **, *P*<0.01 compared with mimics NC group. (**D**) Cell apoptosis rate was detected by flow cytometry (annexin V-FITC staining). *, *P*<0.05 and **, *P*<0.01 compared with blank control group. ^##^, *P*<0.01 compared with mimics NC group. (**E**) The activation of AKT was assessed by Western blot. All experiments were performed in triplicate, and data were represented as mean ± S.D.

In order to simulate hepatic I/R injury, we further established an *in vitro* H_2_O_2_-induced apoptosis model. AML-12 cells were treated with H_2_O_2_ (0–400 µM) for 6 h, and *miR-494* mRNA expression was decreased in a dose-dependent manner when compared with control cells without H_2_O_2_ treatment (all *P*<0.05 or 0.01, Figure 3A). Further, AML-12 cells were transfected with *miR-494* mimics or mimics NC, and treated with 200 µM H_2_O_2_ for 6 h at all indicated time points. CCK8 assay showed that *miR-494* mimics significantly improved cell viability compared with mimics NC group (all *P*<0.01, Figure 3B). As shown in Figure 3C,D, the apoptosis rate of *miR-494* mimics was significantly lower than that in mimics NC group (*P*=0.002), and the expression of apoptosis-related proteins cle-PARP and cle-Caspase-3 was also significantly reduced (*P*=0.014 and 0.038, respectively), suggesting that *miR-494* decreased H_2_O_2_-induced apoptosis of hepatic AML12 cells. To elucidate the potential mechanism, we further examined the intracellular level of activated AKT, and found that p-AKT expression in *miR-494* mimics was obviously higher compared with mimics NC group (Figure 3E), indicating that the protective effect of *miR-494* against H_2_O_2_-induced cell apoptosis was mediated through the activation of AKT.

### PTEN is a downstream target gene of *miR-494*

**Figure 4:**
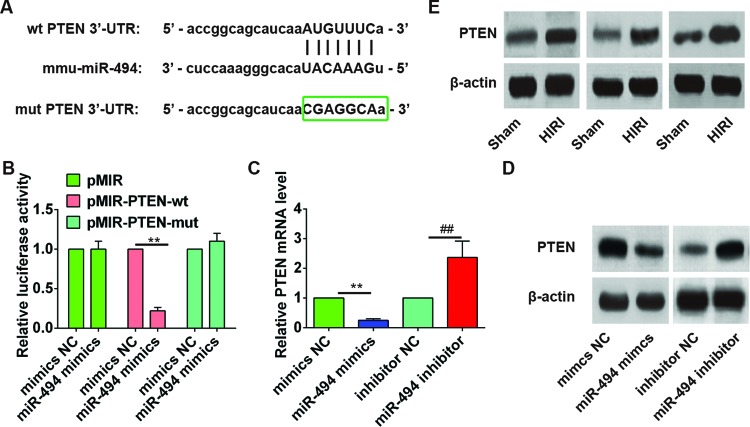
PTEN is a downstream target gene of *miR-494* (**A**) *miR-494*-binding sequences in the 3′-UTR of PTEN and mutated sequence of PTEN 3′-UTR. (**B**) AML12 hepatocytes were cotransfected with PTEN 3′-UTR luciferase constructs (pMIR, pMIR-PTEN-wt or pMIR-PTEN-mut) and mimics NC or *miR-494* mimics for 48 h. Cells were then collected and luciferase activity was determined. The level of firefly luciferase activity was normalized to *Renilla* luciferase activity. *miR-494* suppressed the luciferase activities of the construct containing the 3′-UTR of PTEN. **, *P*<0.01 compared with mimics NC. (**C**,**D**) AML12 cells were transfected with mimics NC, miR-494 mimics, inhibitor NC, and miR-494 inhibitor for 48 h. The expression of *PTEN* mRNA and protein was detected by qRT-PCR and Western blot, respectively. All experiments were performed in triplicate, and data were represented as mean ± S.D. **, *P*<0.01 compared with mimics NC. ^##^, *P*<0.01 compared with mimics NC group. (**E**) Representative images of Western blot analysis comparing PTEN expression in liver samples in sham surgery and HIRI groups (*n*=6).

To identify the downstream target gene of *miR-494*, bioinformatics analyses (PicTar and TargetScan) were performed. PTEN was identified as one of the candidate targets of *miR-494* (Figure 4A). To validate this prediction, the 3′-UTR region of PTEN was then cloned into a luciferase system. It was found that *miR-494* mimics significantly suppressed the luciferase activity of the 3′-UTR wild-type of PTEN (*P*=0.006), but not that of the pMIR-PTEN-mut or pMIR group, when compared with the mimics NC group (Figure 4B). Further, the expression of *PTEN* mRNA in AML12 cells treated with *miR-494* mimics or *miR-494* inhibitor was compared by qRT-PCR. As shown in Figure 4C, *PTEN* mRNA expression was significantly reduced by *miR-494* mimics (*P*=0.008) and enhanced by *miR-494* inhibitor (*P*=0.000). Consistently, PTEN protein expression was also obviously down-regulated after *miR-494* mimics transfection and up-regulated after *miR-494* inhibitor transfection (Figure 4D). Moreover, Western blot revealed that the HIRI rats with lower hepatic *miR-494* expression had obviously higher PTEN level in the liver when compared with sham-operated rats, indicating a negative association between *miR-494* level and PTEN expression (Figure 4E). All these findings confirmed PTEN as the downstream target gene of *miR-494*.

### The protective effect of *miR-494* in HIRI rats is associated with PI3K/AKT signaling pathway

**Figure 5:**
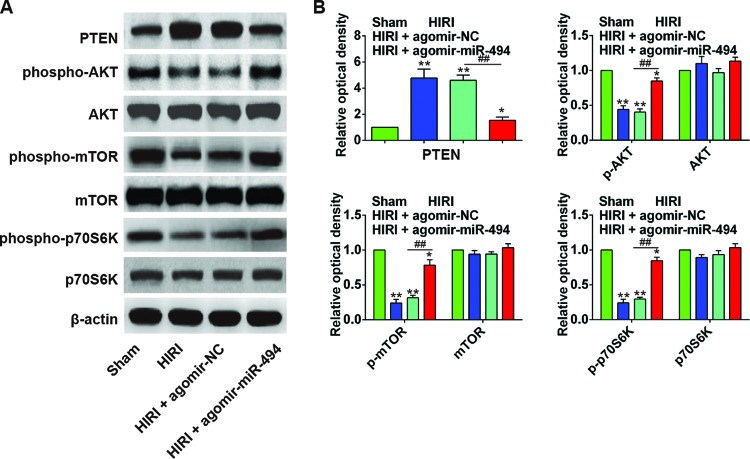
*miR-494* protected liver I/R injury in rats through activating PTEN/PI3K/AKT signaling pathway (**A**,**B**) Western blot analysis comparing PTEN, p-AKT, AKT, p-mTOR, mTOR, p-p70S6K, and p70S6K expression in sham surgery, HIRI, HIRI + agomir-NC, HIRI + agomir-*miR-494* groups (*n*=6); *, *P*<0.05 and **, *P*<0.01 compared with sham surgery group. ^##^, *P*<0.01 compared with agomir-NC group.

To further explore the molecular mechanism underlining the protective effect of *miR-494* in HIRI rats, we examined the expression of proteins in rat liver, including PTEN, p-AKT, AKT, and the downstream effectors of PI3K/AKT pathway (p-mTOR, mTOR, p-p70S6K, and p70S6K). As shown in Figure 5A,B, the HIRI rats had significantly increased PTEN level in the liver, but decreased p-AKT, p-mTOR, and p-p70S6K compared with sham group (all *P*<0.01). After agomir-*miR-494* treatment, the PTEN level in rat liver was significantly inhibited, and p-AKT, p-mTOR, and p-p70S6K were increased compared with agomir-NC group (*P*<0.05 or 0.01), indicating that *miR-494* protected rat liver against I/R injury through activating the PI3K/AKT signaling pathway.

## Discussion

Hepatic I/R injury is one of the major problems during liver surgery, and it greatly affects the surgical outcome(s). Liver I/R injury triggers a rapid release of ROS, leading to hepatocyte injury and apoptosis [19,20]. In the present study, we successfully constructed a rat model of liver I/R injury as indicated by the elevated serum levels of ALT, AST, LDH, and GLDH enzymes, enhanced concentration of hepatic oxidative parameters MDA, TOA, and OSI, extensive hepatocyte necrosis, and increased rate of hepatocyte apoptosis. Differentiated expression of several miRNAs in hepatic I/R injury has been previously reported. For instance, *miR-223* was strongly up-regulated in hepatic IRI, whereas *miR-122* and *miR-146a* were markedly down-regulated [11–13]. In the present study, we found that *miR-494* in HIRI group was significantly down-regulated by at least two fold when compared with sham surgery group. To analyze the regulatory role of *miR-494* in hepatic I/R injury, we treated the HIRI rats with an intraperitoneal injection of agomir-*miR-494* or agomir-NC, and compared the pathophysiological changes in the liver in the two groups. It was found that agomir-*miR-494* significantly decreased serum ALT, AST, LDH, and GLDH levels, liver MDA, TOA, and OSI concentration, hepatocyte apoptosis rate, liver expression of apoptosis-associated proteins (cleaved PARP, cleaved caspase-3, and Bax), hepatocyte necrosis and DNA fragment, when compared with agomir-NC rats. These results suggested that *miR-494* effectively alleviated the liver I/R injuries.

The generation of ROS is an essential factor leading to hepatic I/R injury [21,22]. Therefore, cell models with H_2_O_2_-induced oxidative stress have been commonly used to simulate the hepatic I/R injury [14]. In the present study, we further established an *in vitro* oxidative stress model by H_2_O_2_ treatment, and found that the expression of *miR-494* in hepatic AML12 cells was dose-dependent decrease. We also showed that transfection of *miR-494* mimics significantly improved the proliferation, and reduced apoptosis rate of H_2_O_2_-treated AML12 cells, when compared with mimics NC transfection, indicating that *miR-494* exerted protective effects against H_2_O_2_-induced oxidative stress. The PI3K/AKT/mTOR pathway is an important intracellular signaling pathway in the regulation of cell cycle [23]. Studies have shown that the activation of AKT pathway can significantly reduce H_2_O_2_-induced cell apoptosis [24–26]. In our study, we found that *miR-494* up-regulated the phosphorylation level of AKT in AML12 cells. Moreover, the activity of apoptosis-associated proteins PARP and caspase-3 was decreased after transfection of *miR-494* mimics, suggesting that *miR-494* reduced the H_2_O_2_-induced apoptosis of AML12 cells via activating the AKT pathway.

To locate the target gene of *miR-494*, we searched the miRNA target prediction databases PicTar and TargetScan. PTEN was identified as one of the candidate targets of *miR-494*. Subsequent luciferase reporter assay also confirmed that *miR-494* specifically targetted 3′-UTR of *PTEN* gene. Subsequent *in vitro* cell experiments showed that *PTEN* mRNA and protein expression were markedly down-regulated after *miR-494* mimics transfection and up-regulated after *miR-494* inhibitor transfection, confirming PTEN as a downstream target of *miR-494*. As a natural inhibitor of the PI3K/AKT pathway, PTEN inhibits the phosphorylation of AKT, and plays an important role in the regulation of cell apoptosis [27,28]. Previous studies have shown that *miR-494* can specifically target PTEN, leading to the activation of the PI3K/AKT pathway during various pathophysiologic processes including cell apoptosis, tumor metastasis, and angiogenesis [18,29,30]. The cardioprotective effects of *miR-494* against I/R-induced myocardium injury are also dependent on Akt activation [17]. We therefore hypothesized that *miR-494* might inhibit the expression of PTEN, which activates the PI3K/AKT pathway and reduces the H/R-induced hepatic apoptosis. To test this hypothesis, we analyzed the activity of PTEN and PI3K/AKT pathway, and found that the HIRI rats had significantly increased PTEN level in the liver, but decreased p-AKT, p-mTOR, and p-p70S6K compared with sham group. After agomir-*miR-494* treatment, the PTEN level in rat liver was significantly inhibited, and p-AKT, p-mTOR, and p-p70S6K was increased compared with agomir-NC group (*P*<0.05 or 0.01). Taken together, *miR-494* protected rat liver against I/R injury through activating the PI3K/AKT signaling pathway.

In conclusion, our study showed that *miR-494* protected rats against hepatic I/R injury through down-regulating its downstream target gene *PTEN*, leading to the activation of PI3K/AKT signaling pathway. Our finding may contribute to the development of novel therapies for hepatic H/R injuries.

## Abbreviations

ALT: alanine aminotransferase
AST: aspartate aminotransferase
DMEM: Dulbecco's modified Eagle medium
GLDH: glutamate dehydrogenase
HE: hematoxylin and eosin
I/R: ischemia/reperfusion
LDH: lactate dehydrogenase
mut: mutant type
NC: negative control
qRT-PCR: quantitative reverse-transcription PCR
RLA: relative luciferase activity
ROS: reactive oxygen species
SD: Sprague–Dawley
TUNEL: terminal deoxynucleotidyl transferase mediated dUTP nick-end labeling
wt: wild type
